# An Updated Meta-Analysis of Randomized Controlled Trials Assessing the Effect of Sorafenib in Advanced Hepatocellular Carcinoma

**DOI:** 10.1371/journal.pone.0112530

**Published:** 2014-12-02

**Authors:** Songlin Peng, Yang Zhao, Feng Xu, Changjun Jia, Yongqing Xu, Chaoliu Dai

**Affiliations:** Department of Hepatobiliary Surgery, The Affiliated Shengjing Hospital of China Medical University, Shenyang 110004, China; Nanjing, China

## Abstract

**Background:**

The efficacy of sorafenib in the treatment of advanced hepatocellular carcinoma (HCC) remains controversial. Therefore, we conducted a meta-analysis to evaluate the efficacy and safety of sorafenib for treating patients with advanced HCC.

**Methods:**

The PubMed, Embase, and Web of Science databases were searched. Eligible studies were randomized controlled trials (RCTs) that assessed sorafenib therapy in patients with advanced HCC. The outcomes included overall survival (OS), time to progression (TTP), overall response rate (ORR), and toxicities. Hazard ratio (HR) and risk ratio (RR) were used for the meta-analysis and were expressed with 95% confidence intervals (CIs).

**Results:**

Seven RCTs, with a total of 3807 patients, were included in this meta-analysis. All patients received sorafenib alone, or with other chemotherapeutic regimens. Pooled estimates showed that sorafenib improved the OS (HR = 0.74, 95% CI: 0.61, 0.90; P = 0.002), or TTP outcomes (HR = 0.69, 95% CI: 0.55, 0.86; P = 0.001). Subgroup analysis revealed that sorafenib was more effective in the patients with an Eastern Cooperative Oncology Group performance status (ECOG PS) of 1–2 (HR = 0.77, 95% CI: 0.60, 1.0; P = 0.05), or macroscopic vascular invasion (MVI), and/or extrahepatic spread (EHS) (HR = 0.65, 95% CI: 0.46, 0.93; P = 0.02), in terms of OS. Patients who received sorafenib did not have a higher ORR (RR = 0.85, 95% CI: 0.65, 1.11; P = 0.10). In addition, there was a slight increase in toxicity in the sorafenib group.

**Conclusion:**

Treatment with sorafenib significantly improved OS and TTP in patients with advanced HCC. Additional large-scale, well-designed RCTs are needed to evaluate the efficacy of sorafenib-based therapy in the treatment of advanced HCC.

## Introduction

Hepatocellular carcinoma (HCC) is the third most common cause of cancer-related deaths among men and the sixth among women [1]. In the west, approximately 30–40% of all HCC patients are diagnosed at an early stage, and might benefit from curative treatments [2], [3], such as partial hepatectomy, radiofrequency ablation (RFA), and percutaneous ethanol injection (PEI). For patients who undergo these procedures, five-year survival rates of 60–70% can be achieved in selected patients [4]. Patients diagnosed at an intermediate stage may obtain limited survival benefit from transarterial chemoembolization (TACE); however, most patients progress to an advanced stage after the initial therapeutic benefit.

Sorafenib is a multi-target tyrosine kinase inhibitor. It acts by blocking the activities of the serine-threnoine kinases Raf-1 and B-Raf, and the receptor tyrosine kinases of the vascular endothelial growth factor receptors [5]–[7], consequently inhibiting tumor-cell proliferation and tumor angiogenesis [5], [6], [8]. Results from the randomized, placebo-controlled phase-3 Sorafenib HCC Assessment Randomized Protocol (SHARP) trial in patients with advanced HCC showed that treatment with sorafenib could significantly prolong overall survival (OS) and time to progression (TTP) [9], and that sorafenib-associated adverse events were tolerable. Median OS in the sorafenib and placebo groups was 10.7 and 7.9 months, respectively, while median time to progression (TTP) was 5.5 and 2.8 months, respectively [9]. Positive outcomes of sorafenib therapy were also observed in the phase-3 Sorafenib Asia-Pacific trial, which was conducted in China, South Korea, and Taiwan [10]; these trials confirmed the benefits of sorafenib therapy in patients with advanced HCC.

However, baseline characteristics may affect the therapeutic efficacy of sorafenib therapy. Patients with differences in disease etiology, tumor burden, Eastern Cooperative Oncology Group performance status (ECOG PS), and the Barcelona Clinic Liver Cancer (BCLC) tumor stage, may respond differently to sorafenib treatment. To identify whether baseline patient characteristics affect the efficacy and safety of sorafenib in the treatment of advanced HCC, we performed a comprehensive meta-analysis to evaluate its effect in general, and in specific subpopulations.

## Methods and Materials

### Literature research and inclusion criteria

We conducted a comprehensive literature search of the PubMed, Embase, and Web of Science databases to recover all relevant records published up to March 16, 2014. The following search items were used: ("sorafenib"[Supplementary Concept] OR "sorafenib"[All Fields]) AND ("carcinoma, hepatocellular"[MeSH Terms] OR ("carcinoma"[All Fields] AND "hepatocellular"[All Fields]) OR "hepatocellular carcinoma"[All Fields] OR ("hepatocellular"[All Fields] AND "carcinoma"[All Fields])). The search was limited to human subjects and randomized controlled trials (RCTs). No language limitations were imposed. We also manually searched the reference lists of RCTs until no additional eligible trials could be identified. Studies that met the following inclusive criteria were considered eligible for this meta-analysis: (1) study design: RCT; (2) study population: adult patients with advanced (unresectable or metastatic) HCC; adequate renal, cardiac, and hematologic function; and a life expectancy of 12 weeks or more; (3) study intervention: patients in the treatment arm received sorafenib or sorafenib-based therapy, whereas patients in the control arm received placebo or placebo-based (without sorafenib) therapy; (4) outcome measures: OS was the primary outcome measure; the secondary outcomes included TTP, ORR, and toxicity.

### Data extraction and quality assessment

Two investigators (Peng and Dai) independently extracted the following data from each study: first author, year of publication, number of patients (intervention/control), performance status, treatment regimen, OS, TTP, ORR, and toxicity. A standardized Excel file was established to collate the data. In case the same trial appeared in different publications, we only chose the most informative article to avoid duplication of information. Disagreements were resolved through discussion and consensus.

The methodological quality of each study was assessed with the Jadad scale [11]. The scale evaluates three criteria, which include randomization (0–2 points), blinding (0–2 points), and dropouts and withdrawals (0–1 point) in the RCT study. A score of 1 point is assigned when a criterion is appropriately described. The scale ranges from 0 to 5 points; studies with a score ≥3 points are considered to be of high quality [12].

### Statistical analyses

All outcomes were expressed in terms of hazard ratio (HR), risk ratio (RR), and 95% confidence intervals (CIs). The heterogeneity of studies was assessed with Cochran's Q chi-square test and *I* ^2^ analysis. Studies with a P value <0.1 or *I* ^2^>50% [13] were considered to have heterogeneity. A fixed-effects model [14] or random-effects model [15] was used to pool the estimates, depending on the absence or presence of heterogeneity. When considerable heterogeneity was present, sensitivity analyses were conducted to identify potential sources. Publication bias was assessed by using the Begg and Egger tests [16], [17]. In some studies, Kaplan-Meier curves were provided instead of HR and 95% CI; in these cases, we used the method described by Tierney to estimate the HR and 95% CI from the Kaplan-Meier curves [18]. A P value <0.05 was judged as statistically significant, except where otherwise specified. All analyses were performed with STATA, version 12.0 (Stata Corporation, College Station, TX, USA).

## Results

### Identification of eligible studies

**Figure 1:**
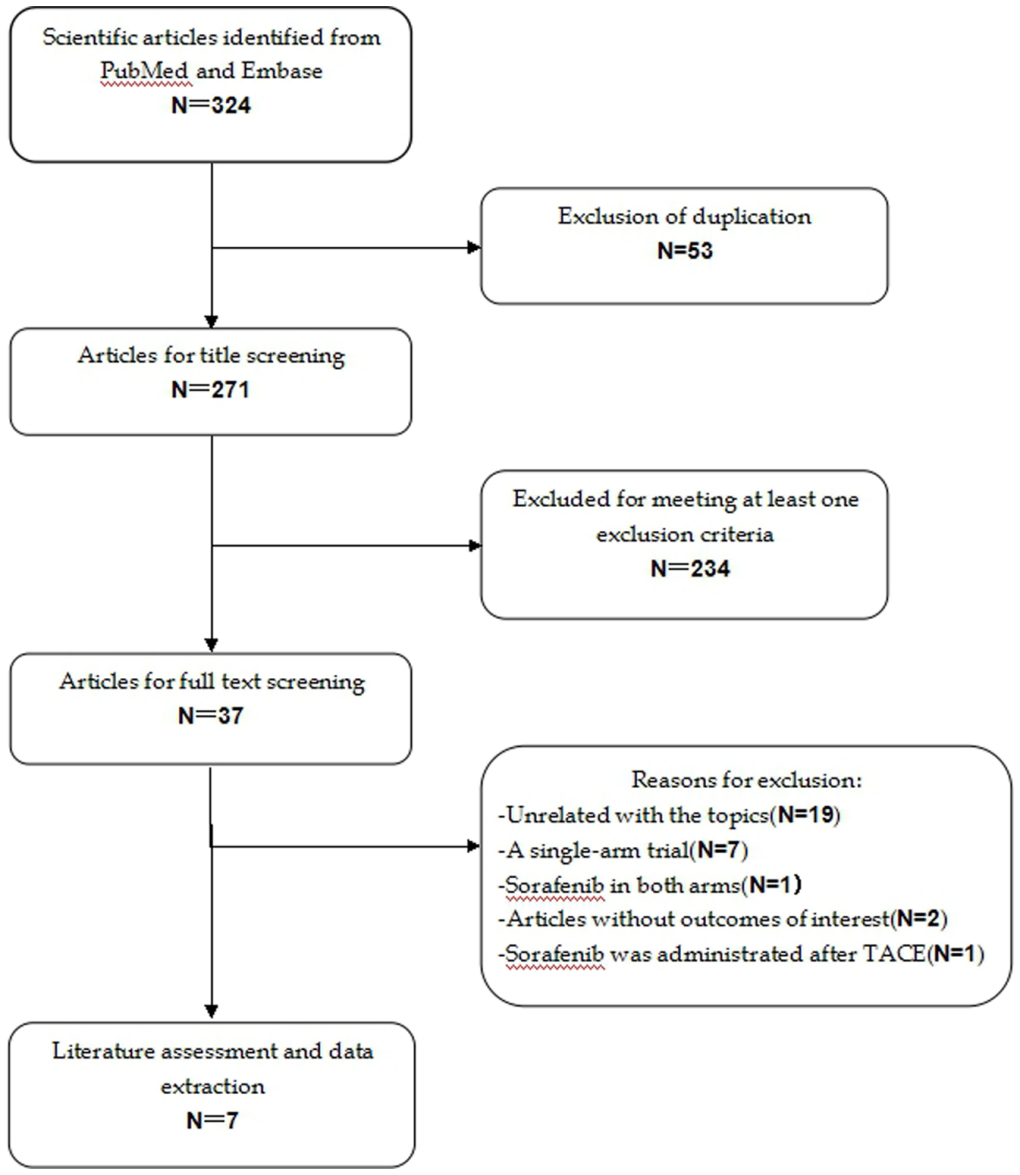
Search strategy and flow chart for the meta-analysis.

The initial database search yielded 324 studies, of which 53 were excluded for duplicate records, and 234 were excluded for various reasons based on the titles/abstracts (Figure 1). The remaining 37 studies were considered for full-text review, of which, the following studies were excluded: two were eliminated as they did not provide any outcomes of interest [19], [20], one assigned sorafenib in both arms [21], seven were single-arm trials [22]–[28], and one included patients who received sorafenib after TACE [29]. Finally, seven RCTs met the inclusion criteria, and were included in this analysis [9], [10], [30]–[34].

### Study characteristics and quality assessment

The main characteristics of the seven RCTs included in this meta-analysis are presented in Table 1. All studies were selected strictly based on prior inclusion criteria. The studies were published between 2008 and 2013. The size of the RCTs ranged from 52 to 1155 patients (total of 3807 patients). The clinical characteristics were well matched for gender, age, status, and stage. Patients in these studies were from Europe, the Americas, Australia, Asia and Africa. The predominant reasons for liver disease were hepatitis C virus infection (44.1%), followed by hepatitis B virus infection (37.7%), and alcohol consumption (14.3%). Approximately 72% of the patients were graded as BCLC stage C, which indicated advanced HCC. Sorafenib was administered alone, or in combination with other chemotherapeutic agents. The dosage and schedule of sorafenib was the one approved by the PDA (400 mg PO twice daily) in each trial. The median Jadad score of the included studies was 4 (range from 4 to 5).

**Table 1:** Baseline characteristics of patients in the trials included in the meta-analysis.

Author	Treatment regimen	No. of patients	Median age (range)	Male/Female	Cause of disease(HBV/HCV/alcohol)	Child-Pugh Class(A/B)	ECOG PS(0/1/2)	BCLC stage(B/C)	MVI/EHS (present/absent)	Jadad score
Bruix J[9]	sorafenib	301	67.5	179/122	32/86/79	190/7	113/84^a^	40/157	133/64	5
	placebo	301	69.0	155/146	28/81/80	187/2	102/87^a^	37/152	125/64	
Cheng AL[10]	sorafenib	150	51(23–86)	127/23	106/16/0	146/4	38/104/8	7/143	54/96	5
	placebo	76	52(25–79)	66/10	59/3/0	74/2	21/51/4	3/73	26/50	
Cheng AL[30]	sorafenib	544	59(18–84)	459/85	288/119/82	No report	288/254/2	89/454	415/129	4
	sunitinib	530	59(18–85)	436/94	290/113/91	No report	278/248/4	67/462	418/112	
Abou-Alfa GK[31]	Doxorubicin+ sorafenib	47	66(38–82)	31/16	3/10/no report	47/0	40^b^/4	No report	No report	4
	Doxorubicin+ placebo	49	65(38–81)	42/7	7/7/no report	47/2	41^b^/3	No report	No report	
Llovet JM[32]	sorafenib	299	64.9±11.2	260/39	56/87/79	284/14	161/114/24	54/244	108/no report	5
	placebo	303	66.3±10.2	264/39	55/82/80	297/6	164/117/22	51/252	123/no report	
Johnson PJ[33]	sorafenib	578	60(25–89)	484/94	258/119/83	531/47	352/226/0	97/449	158/420	4
	Brivanib	577	61(19–87)	483/94	254/116/106	531/46	361/216/0	95/444	155/422	
Rahman OA [34]	sorafenib	26	53.5(33075)	No report	No report	8/18	0/14/12	No report	10/16	4
	capecitabine	26	59.5(42–70)	No report	No report	4/22	0/13/13	No report	7/19	

a, data from ECOG PS 1–2; b, data from ECOG PS 0–1;

ECOG PS, Eastern Cooperative Oncology Group performance status; BCLC, Barcelona Clinic Liver Cancer; MVI, macroscopic vascular invasion; EHS, extrahepatic spread; HBV, hepatitis B virus; HCV, hepatitis C virus.

### Overall survival

**Figure 2:**
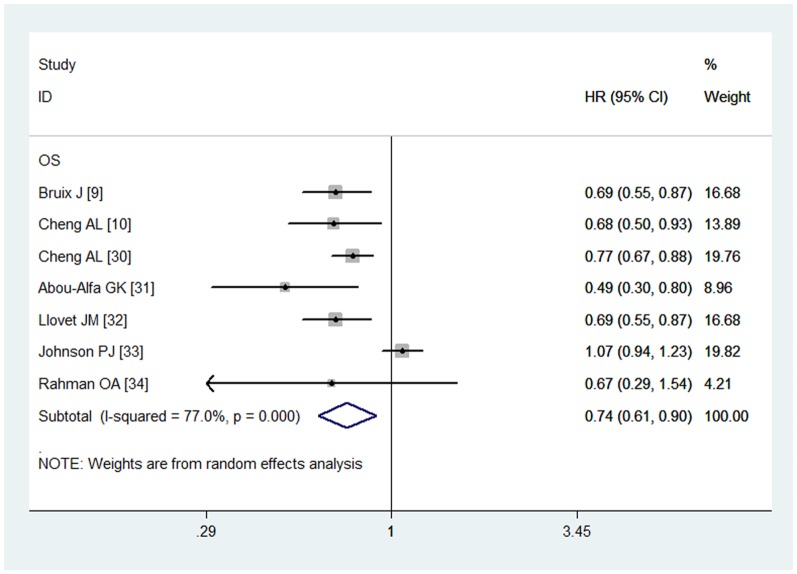
Comparison of sorafenib with other regimens for HCC patients in terms of overall survival (OS).

All the included studies reported the OS data [9], [10], [30]–[34]. The aggregated results suggested that sorafenib was associated with a significant improvement in OS (HR = 0.74, 95% CI: 0.61, 0.90; P = 0.002) (Figure 2). The test for heterogeneity was significant (P = 0.000, *I* ^2^ = 77.0%). Subsequently, we conducted subgroup analysis to explore potential sources of heterogeneity. The results revealed that sorafenib was an effective treatment for patients with ECOG PS of 1–2 (HR = 0.77, 95% CI: 0.60, 1.0; P = 0.05), or macroscopic vascular invasion (MVI) and/or extrahepatic spread (EHS) (HR = 0.65, 95% CI: 0.46, 0.93; P = 0.02) (Table 2). The Begg and Egger tests provided no evidence of publication bias (for Begg's test, Z = 0.75, P = 0.453; for Egger's test, t = 0.28, P = 0.792).

**Table 2:** Summary of subgroup analysis based on baseline prognostic factors.

Patients	OS			TTP		
	HR	95%CI	*P* value	HR	95%CI	*P* value
All	0.74	0.61–0.90	0.002	0.69	0.55–0.86	0.001
HBV-positive	0.91	0.76–1.08	0.267	0.74	0.48–1.14	0.174
HBV-negative	0.86	0.44–1.78	0.732	—	—	—
HCV-positive	0.83	0.32–2.15	0.695	—	—	—
ECOG PS 0	0.81	0.59–1.11	0.193	0.64	0.35–1.18	0.000
ECOG PS 1-2	0.77	0.60–1.00	0.050	0.58	0.44–0.75	0.000
MVI and/or EHS present	0.65	0.46–0.93	0.016	0.44	0.28–0.69	0.000
MVI or EHS absent	0.69	0.46–1.05	0.085	0.62	0.50–0.77	0.000
Normal AFP	0.90	0.48–1.71	0.757	—	—	—
Elevated AFP	0.84	0.54–1.32	0.449	—	—	—

OS, overall survival; TTP, time to progression; HR, hazard ratio; ECOG PS, Eastern Cooperative Oncology Group performance status; BCLC, Barcelona Clinic Liver Cancer; MVI, macroscopic vascular invasion; EHS, extrahepatic spread; HBV, hepatitis B virus; HCV, hepatitis C virus

These pooled results were calculated from the included studies of reference of 9,10,32,33.

### Time to progression

**Figure 3:**
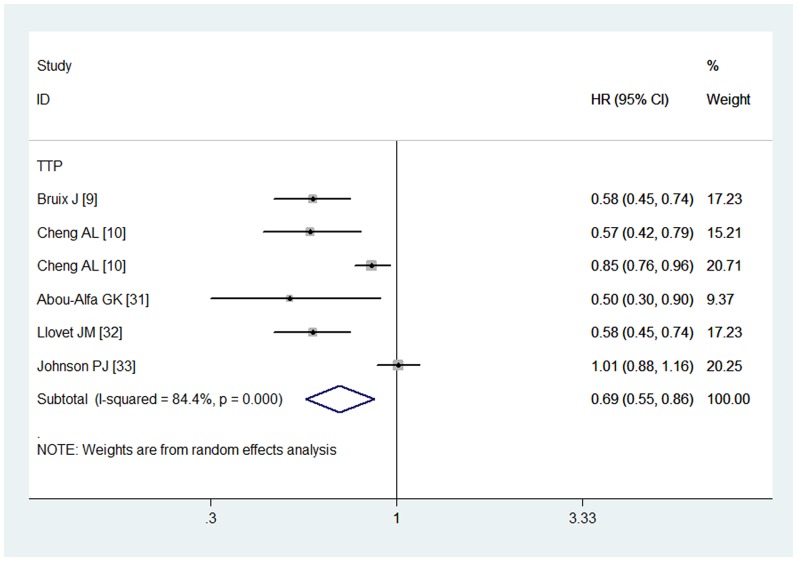
Comparison of sorafenib with other regimens for HCC patients in terms of time to progression (TTP).

Six studies reported the data in terms of TTP [9], [10], [30]–[33]. The pooled estimates using a random-effects model showed that, in advanced HCC, a TTP benefit existed in the sorafenib group when compared with the control group (HR = 0.69, 95% CI: 0.55, 0.86; P = 0.001) (Figure 3). The test for heterogeneity was significant (P = 0.000, *I* ^2^ = 84.4%). Subsequently, we conducted subgroup analysis to explore potential sources of heterogeneity. The results showed significant TTP benefits of sorafenib treatment in the patients irrespective of MVI, EHS, and ECOG status (Table 2). The Begg and Egger tests provided no evidence of publication bias (for Begg's test, Z = 0.19, P = 0.851; for Egger's test, t = 1.06, P = 0.349).

### Overall response rate

**Figure 4:**
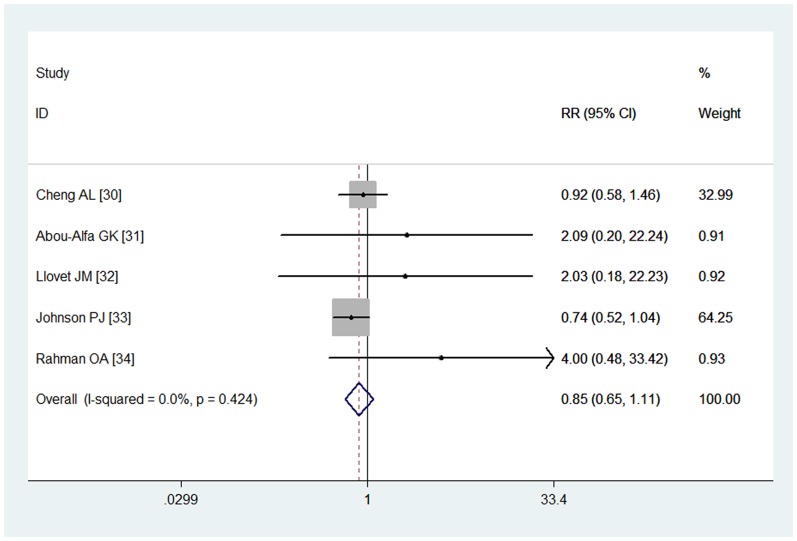
Comparison of sorafenib with other regimens for HCC patients in terms of overall response rate (ORR).

Five studies reported data for ORR [30]–[34]. The pooled results showed that patients treated with sorafenib did not have a higher ORR when compared with other treatments (RR = 0.85, 95% CI: 0.65, 1.11; P = 0.10) (Figure 4). The Begg and Egger tests provided no evidence of publication bias (for Begg's test, Z = 0.73, P = 0.462; for Egger's test, t = 2.01, P = 0.249).

### Adverse events

Five studies reported adverse events [30]–[34]; Table 3 lists the most frequently observed grade 3 or 4 adverse events. The pooled results suggest that sorafenib induced a significantly higher rate of hand-foot syndrome (RR = 5.4, 95% CI: 1.8, 16.2; P = 0.003), diarrhea (RR = 1.45, 95% CI: 1.21, 2.34; P = 0.003), fatigue (RR = 1.70, 95% CI: 1.30, 2.23; P = 0.000), and rash (RR = 3.21, 95% CI: 1.65, 6.26; P = 0.001).

**Table 3:** Summary of the risk ratio (RR) of adverse events in patients with HCC.

Adverse events	Risk ratio (RR)	95% CI	*P* value
Diarrhea	1.45	1.21–2.34	0.003
Hand-foot syndrome	5.40	1.8–16.2	0.003
Rash	3.21	1.65–6.26	0.001
Fatigue	1.70	1.30–2.23	0.000
Hypertension	0.67	0.32–1.42	0.299
Nausea	0.73	0.22–2.38	0.595
Vomiting	0.57	0.19–1.68	0.308
Neutropenia	0.28	0.02–3.60	0.328
Leukopenia	0.82	0.004–164.09	0.942

These pooled results were calculated from the included studies of reference of 30–34.

## Discussion

The main aim of this meta-analysis was to assess the efficacy and safety of sorafenib in patients with advanced HCC. This meta-analysis suggests that while sorafenib significantly improved OS (HR = 0.74, 95% CI: 0.61, 0.90; P = 0.002), and TTP (HR = 0.69, 95% CI: 0.55, 0.86; P = 0.001), it did not increase the ORR (RR = 0.85, 95% CI: 0.65, 1.11; P = 0.10). Moreover, upon subgroup analysis, sorafenib significantly prolonged TTP in patients, irrespective of MVI, EHS, and ECOGPS status. Similarly, an OS benefit was observed in patients with ECOG PS of 1–2 (HR = 0.58, 95% CI: 0.44, 0.75; P = 0.000), or MVI and/or EHS (HR = 0.62, 95% CI: 0.50, 0.77; P = 0.000).

There have been two published meta-analyses of sorafenib therapy for advanced HCC [35], [36]; this study expands on the prior meta-analyses in providing more significant evidence for the use of sorafenib in the treatment of advanced HCC. The larger sample size in our analysis provides a distinct advantage over previous reports in evaluating the efficacy of sorafenib. In addition, all the seven trials included are prospective, randomized controlled phase-3 clinical trials, whereas, in the meta-analysis conducted by Xin Zhang, et al. [35], of the six studies included, three were single-arm phase-2 trials. In order to include the three single-arm trials in the meta-analysis, the authors had introduced the control group from three RCTs as the control arm of these trials [35]; however, despite these modifications to the data from the three single-arm trials, the results revealed no change in the OS and TTP, and the final results may not be reliable. In the present meta-analysis, all included studies were well-designed and of high quality (Jadad score range from 4 to 5); the larger sample size and higher study quality have enabled more accurate and reliable statistical analyses. Furthermore, we also assessed the survival effect of sorafenib in special patients, including those with HBV-positive/negative, ECOG0/1–2, MVI and/or EHS present, normal/elevated AFP, which had not been discussed in the prior meta-analysis.

Among the disease etiologies for HCC, approximately 44% of the patients included in the study had chronic HCV infection, 38% had chronic HBV infection, and 14% suffered from alcohol abuse. It is assumed that chronic viral infections may induce HCC, through mechanisms that differ by specific virus and genotype [37]. Thus, we assume that patients with HBV- or HCV-induced HCC may respond differently to sorafenib treatment. However, in this meta-analysis, patients with both HBV-, and HCV-induced HCC did not receive any significant OS benefit from the sorafenib treatment. Contrary to our findings, in the SHARP trial [9], the authors found that patients with HCV-induced HCC had prolonged median OS (14.0 vs. 7.4 months, HR = 0.50, 95% CI: 0.32, 0.77), whereas patients with HBV-induced HCC did not (HR = 0.76, 95% CI: 0.38, 1.5). In another phase-3 clinical trial [33], the results were consistent with our findings, and neither of the HBV- or HCV- induced HCC patients received OS benefit upon treatment with sorafenib (for HBV-induced HCC, HR = 0.98, 95% CI: 0.80, 1.19; for HCV-induced HCC, HR = 1.33, 95% CI: 0.97, 1.83). However, considering that subgroup analysis was performed on only two phase-3 clinical trials for patients with HBV- or HCV- induced HCC, there is an urgent need for further investigation.

Tumor burden may be defined as MVI and EHS, which are both considered independent factors that affect the mortality of patients with HCC [38]–[40]. The presence of MVI and/or EHS limits the treatment options. For patients with MVI and/or EHS, curative treatment, which may include partial hepatectomy, radiofrequency ablation (RFA), percutaneous ethanol injection (PEI), and transcatheter arterial chemoembolization (TACE), is generally not recommended. In this study, sorafenib significantly prolonged both OS and TTP in patients with or without MVI and/or EHS, compared with other regimens.

In this meta-analysis, we found that the sorafenib-associated adverse events were consistent with those observed in previously published meta-analyses [35], [36]. Grade 3 and 4 adverse events, including hand-foot syndrome (RR = 5.40, 95% CI:1.8, 16.2; P = 0.003), diarrhea (RR = 1.45, 95% CI:1.21, 2.34; P = 0.003), fatigue (RR = 1.70, 95% CI:1.30, 2.23; P = 0.000), and rash (RR = 3.21, 95% CI:1.65, 6.26; P = 0.001), were more commonly observed in the sorafenib group than in the control group. While the sorafenib-associated adverse events were mainly mild to moderate in severity [41], they may lead to dose reduction, or pause in sorafenib treatment. Another problem of note was the risk of hemorrhagic and cardiac events, which has been raised in previous studies [41], [42].

This study had several limitations. First, our meta-analysis is based on seven RCTs; moreover, some of the trials had a relatively small sample size, which might lead to an overestimation of the treatment effect when compared with larger trials. Second, some of our subgroup analyses are based on only 2 to 3 studies; thus, the conclusions on sorafenib efficacy in specific cohorts should be interpreted with caution. Third, there was considerable heterogeneity among the studies, including differences in region, ethnicity, ECOG status, and viral etiology. These factors have the potential to affect our results. Finally, we tried to retrieve confidence intervals data from the investigators; however, this strategy was unsuccessful. Thus, we calculated the values of HRs with 95% CI derived from the Kaplan-Meier curves, which may lead to inaccurate data.

In summary, this study indicated that while sorafenib-therapy prolonged OS and TTP in the patients with advanced HCC, it did not increase ORR. Subgroup analysis showed that sorafenib was more effective in patients irrespective of ECOG PS, or MVI and/or EHS. However, given the limited number of studies included, more prospective RCTs are warranted to evaluate these findings and investigate the efficacy of sorafenib in specific subpopulations of HCC patients.

## Supporting Information

Checklist S1 PRISMA checklist.
(PDF)
